# Altered neuroendocrine control and association to clinical symptoms in adolescent chronic fatigue syndrome: a cross-sectional study

**DOI:** 10.1186/s12967-016-0873-1

**Published:** 2016-05-05

**Authors:** Vegard Bruun Wyller, Valieria Vitelli, Dag Sulheim, Even Fagermoen, Anette Winger, Kristin Godang, Jens Bollerslev

**Affiliations:** Division of Medicine and Laboratory Sciences, Medical Faculty, University of Oslo, Oslo, Norway; Department of Paediatrics, Akershus University Hospital, Nordbyhagen, 1478 Lørenskog, Norway; Department of Biostatistics, Institute of Basic Medical Sciences, Oslo Centre for Biostatistics and Epidemiology, University of Oslo, Oslo, Norway; Department of Paediatrics, Oslo University Hospital, Oslo, Norway; Department of Paediatrics, Lillehammer County Hospital, Lillehammer, Norway; Institute of Clinical Medicine, Medical Faculty, University of Oslo, Oslo, Norway; Department of Anesthesiology and Critical Care, Oslo University Hospital, Oslo, Norway; Institute of Nursing Sciences, Oslo and Akershus University College of Applied Sciences, Oslo, Norway; Section of Specialized Endocrinology, Department of Endocrinology, Oslo University Hospital Rikshospitalet, Oslo, Norway

**Keywords:** Chronic fatigue syndrome, Adolescence, Network analyses

## Abstract

**Background:**

Chronic fatigue syndrome (CFS) is a common and disabling disorder, and a major threat against adolescent health. The pathophysiology is unknown, but alteration of neuroendocrine control systems might be a central element, resulting in attenuation of the hypothalamus–pituitary–adrenalin (HPA) axis and enhancement of the sympathetic/adrenal medulla (SAM) system. This study explored differences in neuroendocrine control mechanisms between adolescent CFS patients and healthy controls, and whether characteristics of the control mechanisms are associated with important clinical variables within the CFS group.

**Methods:**

CFS patients 12–18 years of age were recruited nation-wide to a single referral center as part of the NorCAPITAL project. A broad case definition of CFS was applied. A comparable group of healthy controls were recruited from local schools. A total of nine hormones were assayed and subjected to network analyses using the ARACNE algorithm. Symptoms were charted by a questionnaire, and daily physical activity was recorded by an accelerometer.

**Results:**

A total of 120 CFS patients and 68 healthy controls were included. CFS patients had significantly higher levels of plasma norepinephrine, plasma epinephrine and plasma FT4, and significantly lower levels of urine cortisol/creatinine ratio. Subgrouping according to other case definitions as well as adjusting for confounding factors did not alter the results. Multivariate linear regression models as well as network analyses revealed different interrelations between hormones of the HPA axis, the SAM system, and the thyroid system in CFS patients and healthy controls. Also, single hormone degree centrality was associated with clinical markers within the CFS group.

**Conclusion:**

This study reveals different interrelation between hormones of the HPA axis, the SAM system, and the thyroid system in CFS patients and healthy controls, and an association between hormone control characteristics and important clinical variables in the CFS group. These results add to the growing insight of CFS disease mechanisms.

*Trial registration* Clinical Trials NCT01040429

## Background

Chronic fatigue syndrome (CFS) is characterized by unexplained, long-lasting, disabling fatigue and exertion intolerance, accompanied by pain, cognitive impairments, orthostatic problems and other symptoms [1]. CFS is a major cause of disability among adolescents, and may have detrimental effects on psychosocial and academic development [2, 3], as well as family functioning [4]. Adolescent CFS prevalence is estimated at 0.1–1.0 % [5, 6]. Treatment options are limited.

The pathophysiology of CFS is poorly understood, but several lines of evidence suggest subtle alteration of neuroendocrine control mechanisms. Attenuation of the hypothalamus-pituitary-adrenal axis (HPA axis) is documented in adult [7] as well as adolescent CFS patients [8–10]. Recent findings indicate an association between HPA axis function and the experience of post-exertional malaise (a hallmark of the CFS phenotype) [11], as well as normalization of the HPA axis function during recovery [9, 11]. In addition, previous adolescent studies report enhanced sympathetic cardiovascular nervous activity as well as increased levels of epinephrine and norepinephrine in CFS [10, 12, 13]. The underlying reason for altered autonomic cardiovascular control might be changes in brainstem reflex mechanisms [14]. Again, recovery from clinical symptoms seems to parallel an improvement of sympathetic nervous function [15]. A limited number of studies have addressed thyroid function in CFS; however, Moorkens and co-workers reported increased TSH and normal free thyroxine levels [16]. Taken together, these observations indicate that altered neuroendocrine control mechanisms might be at the core of CFS pathophysiology, in line with contemporary theoretical disease models [17, 18].

Dynamic function tests remain the “gold standard” for exploring neuroendocrine control mechanism, but are often not feasible in large patient cohorts. In addition, such tests often imply infusion of biologically active substances, causing an ethical dilemma in participants that cannot provide fully informed consent. Modern techniques of systems biology, such as network analyses, might provide an alternative approach. Fuite and co-workers demonstrated that adult CFS patients, as compared to healthy controls, display profound re-modeling of neuroendocrine and immune network, suggesting altered control mechanisms [19]. These interesting results from a small-scale exploratory study have to the best of our knowledge never been reproduced in a larger data set, nor have similar analyses been undertaken in adolescent CFS patients.

Thus, the aim of this study was to explore differences in neuroendocrine control mechanisms between adolescent CFS patients and healthy controls by studying the interrelation between hormones of the HPA axis, the sympathetic/adrenal medulla (SAM) system, and the thyroid system in the two groups. Furthermore, we explored whether characteristics of the control mechanisms are associated with important clinical variables among the CFS patients.

## Methods

### CFS patients

The Department of Paediatrics at Oslo University Hospital is a national referral center for young CFS patients. For this study, all hospital paediatric departments in Norway (n = 20), as well as primary care paediatricians and general practitioners, were invited to refer CFS patients aged 12–18 years consecutively to our department. Details of the recruitment procedure are reported elsewhere [10]. Patients considered eligible to this study were summoned to a clinical encounter at our study center after which a final decision on inclusion was made.

In agreement with clinical guidelines [20], we applied a ‘broad’ case definition of CFS, requiring 3 months of unexplained, disabling chronic/relapsing fatigue of new onset. We did not require that patients meet any other accompanying symptom criteria, in contrast to the case definitions from the International Chronic Fatigue Syndrome Study Group at the Centers for Disease Control and Prevention (commonly referred to as the Fukuda-definition) [21], and the Canadian Consensus Criteria (the Canada 2003-definition) [22]. However, the validity of these definitions has not been established [23], and empirical findings raise concerns about the validity, in particular among adolescents [24–26]. In the present study, subgrouping of the participants according to the Fukuda-definition and Canada 2003-definition was performed post hoc, based on questionnaire results (cf. below).

### Healthy controls

A group of healthy controls with a comparable distribution of gender and age were recruited from local schools. Controls were not matched to cases on any variable. No chronic disease and no regular use of pharmaceuticals were allowed.

### Study design and ethics

This study is part of the NorCAPITAL-project (The Norwegian Study of Chronic Fatigue Syndrome in Adolescents: Pathophysiology and Intervention Trial; ClinicalTrials ID: NCT01040429), and details of study design have been provided elsewhere [10]. Briefly, data were collected in the period March 2010 until October 2012. A 1-day in-hospital assessment included clinical examination and blood sampling, and always commenced between 7.30 and 9.30 a.m. All participants were instructed to abstain from tobacco products and caffeine at least 48 h in advance, to fast overnight and to bring a morning spot urine sample in a sterile plastic container. They were also instructed to apply an ointment containing the local anesthetic lidocaine (Emla^®^) on the skin in the antecubital area 1 h prior to the blood sampling. After at least 5 min supine rest in calm surroundings, blood samples were obtained in a fixed sequence from antecubital venous puncture. Following the in-hospital assessment, daily physical activity was monitored during seven consecutive days using an accelerometer, and a self-administered questionnaire was completed. After completion of baseline assessment, the CFS patients were subjected to a randomized controlled trial of low-dose clonidine featuring follow-up controls at week 8 and week 30 [10]; this paper, however, report baseline result only.

NorCAPITAL has been approved by the Norwegian National Committee for Ethics in Medical Research and the Norwegian Medicines Agency. Written informed consent was obtained from all participants, and from parents/next-of-kin if required.

### Questionnaire

In accordance with a reliable and valid CFS symptom inventory for adults [27], we have developed a CFS symptom inventory for adolescents, assessing the frequency of 24 common symptoms during the preceding month [10]. Each symptom is rated on a 5-point Likert scale, ranging from ‘never/rarely present’ to ‘present all of the time’. The inventory includes accompanying symptom of the Fukuda-definition and Canada 2003-defintion, facilitating post hoc subgrouping of CFS patients.

The Chalder Fatigue Questionnaire (CFQ) [28] is regarded a reliable and valid measure in CFS research among adolescents [29]. In this study, the CFQ total sum score is applied (i.e., the sum across all 11 CFQ items, each of which is scored on a 0–3 Likert scale). Total range is from 0 to 33; higher scores imply more severe fatigue.

The Mood and Feelings Questionnaire (MFQ) has been thoroughly validated in children and adolescents [30], and is also shown to have good reliability [31]. MFQ consists of 34 items, each scored on a 0–2 Likert scale; thus, the total sum score is from 0 to 68. A score ≥20 implies presence of depressive symptoms to a degree that suggests a mood disorder.

The questionnaire also charted other relevant variables, such as disease duration (in the CFS group) and menstrual cycle characteristics (in females).

### Daily physical activity

Accelerometers have been successfully applied in previous CFS studies [32, 33]. In this study, we used the *activPAL* accelerometer device (PAL Technologies Ltd, Glasgow, Scotland) which provides inter device reliable and valid data on step number and cadence as well as time spent on walking, standing and sitting/lying during everyday activities [34, 35]. A recording period of seven consecutive days was selected.

Data from the recording units was transferred to a computer running producer developed software. For each participant, all recording epochs were carefully and independently reviewed by two of the authors (DS and EF), and the mean number of steps per day was calculated for all recording epochs. Details on the activity recording procedure have been reported elsewhere [10].

### Laboratory assays

The blood samples for plasma norepinephrine (NE) and epinephrine (E) analyses were obtained in vacutainer tubes treated with ethylene glycol tetraacetic acid (EGTA)–Glutathione. The samples were placed on ice for approximately 30 min; thereafter, plasma was separated by centrifugation (3000 rpm, 15 min, 4 °C) and frozen at −80 °C until assayed. Samples were analyzed for plasma NE and E by high-performance liquid chromatography (HPLC) with a reversed-phase column and glassy carbon electrochemical detector (Antec, Leyden Deacade II SCC, Zoeterwoude, The Netherlands) using a commercial kit (Chromsystems, München, Germany) [36, 37]. The intra- and interassay coefficient of variation (CV) were 3.9 and 10.8 %, respectively. The detection limit was 5.46 pm. Plasma cortisol as well as plasma levels of adrenocorticotrophic hormone (ACTH), thyroid-stimulating hormone (TSH), and free thyroxine (FT4) were determined by routine assays at the accredited laboratory at Oslo University Hospital, Norway.

Urine samples for NE and E analyses were acidified to pH 2.5 immediately after collection, and thereafter stored at 2–8 °C until assayed. Urine treated this way is stable at least 5 days. The same HPLC protocol as for plasma measurement was used for the measurement of urin NE/E. The intra- and interassay coefficient of variation (CV) for urine were 3.9 and 5.2 %, respectively. For determination of urine free cortisol (non-conjugated cortisol), the urine samples were extracted with ether to avoid interference from other steroids, and thereafter assayed by solid phase competitive luminescence immunoassay (LIA) (type Immulite^®^ 2000, Siemens Healthcare Diagnostics, NY, USA) [38]. Intra- and interassay CV were <10 %. The urine levels of creatinine were analyzed using standard automatic analyzer techniques at the accredited laboratory at Oslo University Hospital, Norway. All urine analyses were performed consecutively.

### Statistical analysis

As the CFS patients were included in a randomised controlled trial, individual data from follow-up consultations (when available) were used for imputation of missing data at baseline. For the remaining missing data (appr. 1 % of total) we used single imputation, as the results aggregation step required by a multiple imputation procedure would be challenging in the context of network analysis (cf. below), and only marginally improve the efficiency of the estimation procedure [39].

Statistical analyses were carried out in SPSS (SPSS Inc., Chicago, Illinois, USA) and R [40]. Patients with CFS were compared with healthy controls by applying Student t, Mann–Whitney, χ^2^, or Fisher exact tests as appropriate. CFS patients adhering to the Fukuda-definition and the Canada 2003-definition were compared to the healthy controls in the same way. Multivariate linear regression analyses were applied to adjust across-group p values for the possible confounding effects of gender, age, BMI and depressive symptoms. The level of significance was set at 0.05.

Relationships between hormones were first explored in separate multivariate linear regression models for CFS patients and healthy controls, respectively, and thereafter by network analyses in the two groups (cf. below). As previous studies have been mainly concerned with altered control of the HPA axis and SAM-system, we focused the analyses on these two systems. The across-group comparisons of network parameters imply a large number of statistical tests, requiring adjustment of the significance level according to the Bonferroni method.

The networks of associations among hormones for cases and controls were estimated separately using the ARACNE algorithm [41, 42], as implemented in the R package bnlearn [43]. All measured hormone levels (a total of nine) were considered *nodes* in the network. To define the network parameters, let G = (V, E) be a graph, with nodes (vertices) V and links (edges) E. Hence, a link between two nodes in the graph describes an association between the corresponding hormone levels. Network parameters were computed for each of the nine nodes in the network using the R package igraph [44]. The *degree* of a node $v \in V$ is the number of links incident upon $v$, and we denote it $C_{D} \left( v \right) = \deg \left( v \right)$. The *closeness*, $C_{C} \left( v \right)$, of *v* is the inverse of its farness, where the latter measures the sum of its distances to all other nodes. *Betweenness*, $C_{B} \left( v \right)$, measures the number of times the shortest path between two other nodes goes through v. Finally, the *eigenvector centrality* of each node, $C_{E} \left( v \right)$, was computed. These network parameters are defined for each node, but a global measure of the corresponding parameter for all nodes in the network can be also computed using the concept of centralization. In other words, aggregate measures for an entire endocrine network can be found, as has previously been done for immune markers and described in detail elsewhere [45]. A bootstrap procedure was applied in order to estimate a confidence interval for each centralized network parameter. These centralized parameters were recomputed 10, 000 times on the networks estimated through subsampling, separately for cases and controls. To ensure coherence of the whole procedure, the subsample size was held equal to the number of samples in each bootstrapping replication. The centralization measures were computed for each network estimated in each bootstrapping run, so that confidence intervals for the differences in centralized network parameters between cases and controls could be computed.

Fuite and co-workers reported that estimated endocrine networks for cases and controls can be similar in overall connectivity but visibly different in topology; i.e., that the distribution of centrality among the nodes within each network is markedly different [19]. This means that a global measure of centrality might not be a reliable parameter, and we therefore also analysed single node centrality across groups. We again performed bootstrapping in order to derive an estimate of the variability and construct confidence intervals, and performed a t test to assess the significance of the across-group difference for each node. Finally, in order to explore the possible relationship between network characteristics and clinical features, the single node degree centrality for CFS patients having Chalder fatigue score above or at median (median = 20) were compared to those having score below median. Likewise, patients having steps/day above or at median (median = 4293) were compared to those below median.

## Results

A total of 120 CFS patients and 68 healthy controls were included. CFS patients had significantly higher scores for depressive symptoms and fatigue, and lower number of steps per day as compared with healthy controls (Table 1). Gender, age, body mass index and menstrual characteristics were similarly distributed in the two groups. In the CFS group, 75 % adhered to the Fukuda definition, and 40 % adhered to the Canada 2003-definition.

**Table 1:** Background characteristics

	CFS patients	Healthy controls	P value
Count—no.	120	68	
Gender—no. (%)			
Male	32 (27)	22 (32)	0.408
Female	88 (73)	46 (68)	
Ethnicity—no. (%)			
Scandinavian	118 (98)	62 (91)	0.027
Not Scandinavian	2 (1.7)	6 (8.8)	
Age—years, mean (SD)	15.4 (1.6)	15.1 (1.6)	0.179
Body mass index—kg/m^2^, mean (SD)	21.5 (4.2)	20.6 (3.7)	0.131
Experienced menarche (females only)—no. (%)			
No	11 (13)	5 (19)	0.411
Yes	75 (87)	21 (81)	
Days since last menstrual bleeding (females only)—median (IQR)	15 (15)	16 (14)	0.884
Disease duration—months, median (IQR)	18 (14)	n.a.	n.a.
Depressive symptom score—mean (SD)	17 (10)	6 (8)	<0.001
Fatigue score—mean (SD)	19 (6)	9 (5)	<0.001
Steps per day—mean (SD)	4662 (2386)	10,293 (3716)	<0.001
Adherence to CDC diagnostic criteria—no. (%)			
No	29 (25)	n.a.	n.a.
Yes	88 (75)	n.a.	
Adherence to Canada diagnostic criteria—no. (%)			
No	69 (60)	n.a.	n.a.
Yes	46 (40)	n.a.	

*CFS* chronic fatigue syndrome, *SD* standard deviation, *IQR* interquartile range, *n.a.* not applicable

CFS patients had significantly higher levels of plasma norepinephrine (p < 0.001), plasma epinephrine (p = 0.002) and plasma FT4 (p = 0.008), and significantly lower levels of urine cortisol/creatinine ratio (p = 0.001) (Table 2). Urine norepinephrine/creatinine ratio was slightly higher in the CFS group. P values were not substantially affected when adjusting for the possible confounding effects of gender, age, BMI and depressive symptoms. Also, separate comparisons of the Fukuda- and Canada 2003-subgroups of CFS patients with healthy controls did not reveal any substantial effect of subgrouping (Table 3).

**Table 2:** Hormone levels among CFS patients and healthy controls

	CFS patients	Healthy controls	P value	P value, adjusted for gender, age, BMI, and depressive symptoms^a^
Plasma norepinephrine—mean (SD)	1981 (777)	1497 (418)	*<0.001*	*<0.001*
Urine norepinephrine:creatinine ratio—median (IQR)	12.4 (5.8)	10.6 (5.8)	0.075	*0.01*
Plasma epinephrine—median (IQR)	308 (130)	267 (99)	*0.002*	*<0.001*
Urine epinephrine:creatinine ratio—median (IQR)	1.25 (1.22)	1.45 (0.99)	0.688	0.887
Plasma ACTH—median (IQR)	3.80 (2.70)	4.07 (2.90)	0.272	0.368
Plasma cortisol—mean (SD)	365 (145)	351 (149)	0.536	0.792
Urine cortisol:creatinine ratio—median (IQR)	3.45 (3.25)	5.34 (2.76)	*0.001*	*0.002*
Plasma TSH—mean (SD)	2.63 (1.08)	2.76 (1.43)	0.497	0.703
Plasma FT4—mean (SD)	15.4 (2.2)	14.6 (1.8)	*0.008*	*0.015*

Italic values indicate significance of p value (p < 0.05)

*CFS* chronic fatigue syndrome, *SD* standard deviation, *IQR* interquartile range, *ACTH* adrenocorticotrophic hormone, *TSH* thyroid stimulating hormone, *FT4* free thyroxine

^a^Applying multivariate linear regression modelling. In order to obtain an approximate normal distribution for all dependent variables, urine norepinephrine:creatinine ratio and urine cortisol:creatinine ratio was ln-transformed, and three extreme outliers for plasma epinephrine were removed and replaced by imputed values

**Table 3:** Hormone levels among subgroups of CFS patients as compared with healthy controls

	All CFS patients	Patients adhering to Fukuda- definition (n = 88)	Patients adhering to Canada 2003-definition (n = 46)	Healthy controls	P value Fukuda vs healthy controls	P value Canada 2003 vs healthy controls
Plasma norepinephrine—mean (SD)	1981 (777)	1964 (806)	1999 (906)	1497 (418)	*<0.001*	*0.001*
Urine norepinephrine:creatinine ratio—median (IQR)	12.4 (5.8)	12.3 (5.0)	13.1 (5.3)	10.6 (5.8)	0.088	*0.029*
Plasma epinephrine—median (IQR)	308 (130)	309 (130)	304 (142)	267 (99)	*0.004*	*0.019*
Urine epinephrine:creatinine ratio—median (IQR)	1.25 (1.22)	1.23 (1.10)	1.27 (1.10)	1.45 (0.99)	0.466	0.789
Plasma ACTH—median (IQR)	3.80 (2.70)	3.70 (2.70)	4.35 (2.60)	4.07 (2.90)	0.272	0.822
Plasma cortisol—mean (SD)	365 (145)	360 (143)	383 (153)	351 (149)	0.726	0.268
Urine cortisol:creatinine ratio—median (IQR)	3.45 (3.25)	3.47 (3.30)	3.44 (2.80)	5.34 (2.76)	*0.003*	*0.005*
Plasma TSH—mean (SD)	2.63 (1.08)	2.48 (0.96)	2.67 (0.87)	2.76 (1.43)	0.17	0.695
Plasma FT4—mean (SD)	15.4 (2.2)	15.4 (2.3)	15.6 (2.5)	14.6 (1.8)	*0.013*	*0.027*

Italic values indicate significance of p value (p < 0.05)

*CFS* chronic fatigue syndrome, *SD* standard deviation, *IQR* interquartile range, *ACTH* adrenocorticotrophic hormone, *TSH* thyroid stimulating hormone, *FT4* free thyroxine

**Fig. 1:**
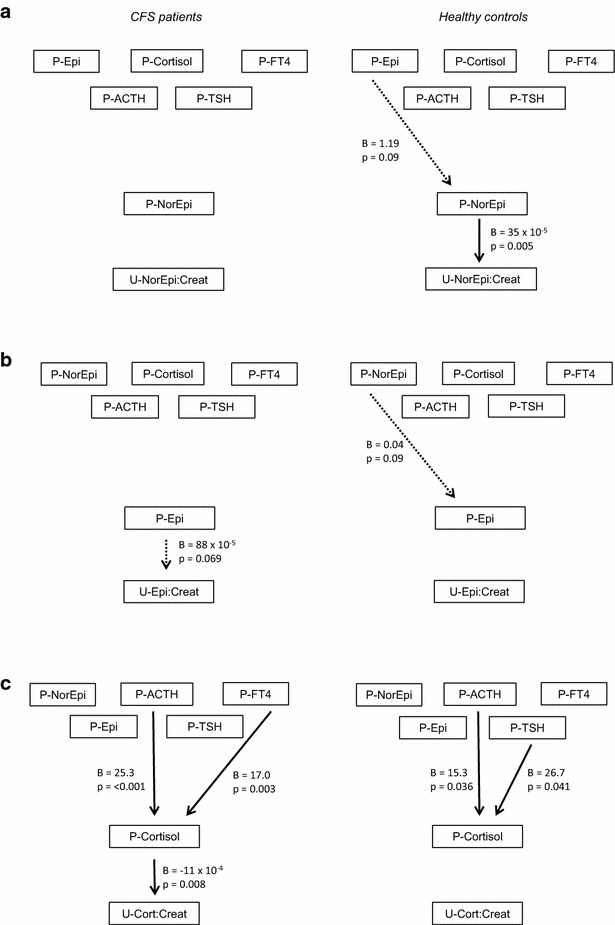
Results of multivariate linear regression modelling in CFS patients (*left*) and healthy controls (*right*). **a** Plasma norepinephrine and urine norepinephrine:creatinine as dependent variables. **b** Plasma epinephrine and urine epinephrine:creatinine as dependent variables. **c** Plasma cortisol and urine cortisol:creatinine as dependent variables. *P* plasma, *U* urine, *Epi* epinephrine, *FT4* free thyroxine, *ACTH* adrenocorticotrophic hormone, *TSH* thyroid stimulating hormone, *NorEpi* norepinephrine, *Cort* cortisol, *Creat* creatinine

In healthy controls, urine norepinephrine:creatinine was significantly associated with plasma norepinephrine in multivariate linear regression models (Table 4; Fig. 1a). In CFS patients, no such association was found. However, the CFS group displayed a weak association between urine epinephrine:creatinine and plasma epinephrine which was not seen in healthy controls (Table 4; Fig. 1b). Also, in CFS patients, urine cortisol:creatinine was strongly associated with plasma cortisol, which in turn was associated with both plasma ACTH and plasma FT4 (Table 4; Fig. 1c). In healthy controls, there was no significant relationship between urine cortisol:creatinine and plasma cortisol. Furthermore, plasma cortisol was not associated with plasma FT4 and the association to plasma ACTH was attenuated as compared with the CFS group; instead, an association between plasma cortisol and plasma TSH was found.

**Table 4:** Relationship between selected markers of sympathetic and HPA activity, and other hormones

	CFS patients	Healthy controls
Dependent variable: plasma norepinephrine		
R squared	0.015	0.043
Plasma epinephrine		
Regression coefficient, B (95 % CI)	0.82 (−0.38 to 2.02)	1.19 (−0.19 to 2.58)
p value	0.179	*0.090*
Dependent variable: urine norepinephrine:creatinine ratio		
R squared	0.009	0.114
Plasma norepinephrine		
Regression coefficient, B (95 % CI)	4.8 (−4.6 to 14) × 10^−5^	35 (11–59) × 10^−5^
p value	0.314	*0.005*
Dependent variable: plasma epinephrine		
R squared	0.015	0.043
Plasma norepinephrine		
Regression coefficient, B (95 % CI)	0.02 (−0.01 to 0.05)	0.04 (−0.01 to 0.08)
p value	0.179	*0.090*
Dependent variable: urine epinephrine:creatinine ratio		
R squared	0.028	0.000
Plasma epinephrine		
Regression coefficient, B (95 % CI)	88 (−7 to 182) × 10^−5^	−11 (−221 to 199) × 10^−5^
p value	*0.069*	0.917
Dependent variable: plasma cortisol		
R squared	0.184	0.184
Plasma ACTH		
Regression coefficient, B (95 % CI)	25.3 (13.4–37.2)	15.3 (1.1–29.6)
p value	*<0.001*	*0.036*
Plasma TSH		
Regression coefficient, B (95 % CI)	12.7 (−9.4 to 35.0)	26.7 (1.1–52.4)
p value	0.262	*0.041*
Plasma FT4		
Regression coefficient, B (95 % CI)	17.0 (6.0–28.0)	−4.9 (−24.2 to 14.4)
p value	*0.003*	0.613
Dependent variable: urine cortisol:creatinine ratio		
R squared	0.058	0.016
Plasma cortisol		
Regression coefficient, B (95 % CI)	−11 (−19 to −3.0) × 10^−4^	−7 (−20 to 6.8) × 10^−4^
p value	*0.008*	0.311

Multivariate linear regression models. The final mulitvariat linear regression models for each dependent variable in CFS patients and healthy controls, respectively, cf. Fig. 1

Italic values indicate significance of p value (p < 0.05)

*CFS* chronic fatigue syndrome, *HPA* hypothalamus–pituitary–adrenal, *CI* confidence interval, *ACTH* adrenocorticotrophic hormone, *TSH* thyroid stimulating hormone, *FT4* free thyroxine

**Fig. 2:**
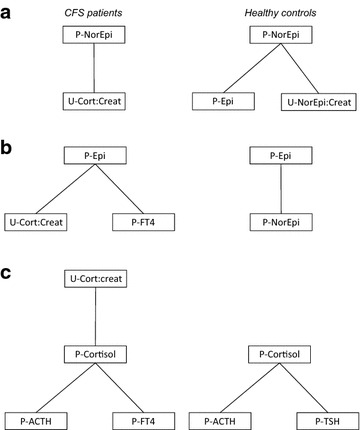
Results of network analyses (single node diagrams) in CFS patients (*left*) and healthy controls (*right*). **a** Plasma norepinephrine as single node. **b** Plasma epinephrine as single node. **c** Plasma cortisol as single node. *P* plasma, *U* urine, *Epi* epinephrine, *FT4* free thyroxine, *ACTH* adrenocorticotrophic hormone, *TSH* thyroid stimulating hormone, *NorEpi* norepinephrine, *Creat* creatinine

Centralized network parameters were equal across the two groups (Table 5). However, all single hormone degree centralities were significantly different across groups (Table 6). Of particular interest, degree centrality for plasma norepinephrine and urine norepinephrine:creatinine was lower among CFS patients as compared with healthy controls, whereas degree centrality for plasma epinephrine, urine epinephrine:creatinine, plasma cortisol and urine cortisol:creatinine was highest in the CFS group (Table 6; Fig. 2).

**Table 5:** Centralized network parameters among CFS patients and healthy controls

	CFS patients	Healthy controls	95 % CI (CFS patients—healthy controls)
Betweenness centrality	0.49	0.61	(−0.29 to 0.46)
Closeness centrality	0.44	0.54	(−0.23 to 0.35)
Degree centrality	0.64	0.69	(−0.39 to 0.50)
Eigenvector centrality	0.50	0.58	(−0.22 to 0.31)

*CFS* chronic fatigue syndrome, *CI* confidence interval

**Table 6:** Single hormone degree centrality (DC) among CFS patients and healthy controls

	CFS patients	Healthy controls	Difference in DC (95 % CI, CFS patients—healthy controls)	p value*
Plasma norepinephrine	0.36	0.44	−0.09 (−0.15 to −0.03)	*0.003*
Urine norepinephrine:creatinine ratio	0.49	1.00	−0.51 (−0.57 to −0.44)	*<0.001*
Plasma epinephrine	0.59	0.17	0.42 (0.36–0.48)	*<0.001*
Urine epinephrine:creatinine ratio	0.72	0.38	0.34 (0.28–0.40)	*<0.001*
Plasma ACTH	0.54	0.55	−0.02 (−0.08 to 0.04)	0.572
Plasma cortisol	0.78	0.46	0.33 (0.26–0.39)	*<0.001*
Urine cortisol:creatinine ratio	1.00	0.63	0.37 (0.31–0.43)	*<0.001*
Plasma TSH	0.36	0.65	−0.29 (−0.35 to −0.24)	*<0.001*
Plasma FT4	0.67	0.63	0.04 (−0.02 to 0.10)	0.231

Italic values indicate significance of p value (p < 0.05)

*CFS* chronic fatigue syndrome, *CI* confidence interval, *ACTH* adrenocorticotrophic hormone, *TSH* thyroid stimulating hormone, *FT4* free thyroxine

* t test

**Fig. 3:**
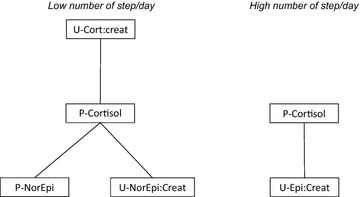
Results of network analyses for plasma cortisol (single node diagram) in CFS patients having low number of step/day (*left*) and high number of step/day (*right*). *P* plasma, *U* urine, *Epi* epinephrine, *NorEpi* norepinephrine, *Cort* cortisol; *Creat* creatinine

Within the CFS group, single hormones were not associated with clinical markers, except for a positive association between urine cortisol:creatinine and steps per day (Table 7). However, as for degree centrality of single hormones, the most disabled patients differed from the least disabled (Table 8). Of particular interest, plasma cortisol degree centrality was highest among those with the highest fatigue score and the lowest number of steps per day (Table 8; Fig. 3).

**Table 7:** Association between single hormones and clinical markers in CFS patients—correlation and regression analyses

	Fatigue score			Steps per day		
	Kendall correlation	Pearson correlation	Bivariate linear regression	Kendall correlation	Pearson correlation	Bivariate linear regression
Plasma norepinephrine						
Coefficient (correlation/regression)	−0.061	−0.078	−0.001	−0.034	−0.002	−0.005
P value	0.345	0.405	0.401	0.588	0.987	0.987
Urine norepinephrine:creatinine ratio						
Coefficient (correlation/regression)	−0.071	−0.105	−1.43	0.096	0.152	871
P value	0.271	0.264	0.296	0.124	0.100	0.107
Plasma epinephrine						
Coefficient (correlation/regression)	−0.075	−0.156	−0.008	0.089	0.117	2.35
P value	0.245	0.095	0.101	0.155	0.209	0.206
Urine epinephrine:creatinine ratio						
Coefficient (correlation/regression)	−0.048	−0.093	−0.885	−0.001	0.011	42
P value	0.455	0.318	0.321	0.983	0.906	0.906
Plasma cortisol						
Coefficient (correlation/regression)	−0.045	−0.066	−0.003	−0.078	−0.081	−1.27
P value	0.480	0.480	0.479	0.213	0.385	0.398
Urine cortisol:creatinine ratio						
Coefficient (correlation/regression)	0.018	0.057	0.358	0.082	*0.242*	*743*
P value	0.785	0.540	0.666	0.190	*0.006*	*0.022*

Italic values indicate significance of p value (p < 0.05)

**Table 8:** Single hormone degree centrality (DC) among the most and the least disabled patients

	Fatigue score				Steps per day			
	High fatigue	Low fatigue	Difference in DC (95 % CI, high–low)	p value*	Low steps/day	High steps/day	Difference in DC (95 % CI, low–high)	p value*
Plasma norepinephrine	0.80	0.48	0.32 (0.25–0.39)	*<0.001*	0.45	0.50	−0.05 (−0.12 to 0.02)	0.140
Urine norepinephrine:creatinine ratio	0.80	0.63	0.17 (0.10–0.25)	*<0.001*	0.89	0.50	0.39 (0.32–0.47)	*<0.001*
Plasma epinephrine	0.45	0.48	−0.04 (−0.11 to 0.04)	0.332	0.45	0.50	−0.05 (−0.12 to 0.02)	0.145
Urine epinephrine:creatinine ratio	0.45	0.48	−0.04 (−0.12 to 0.05)	0.381	1.00	1.00	0.00 (−0.07 to 0.07)	1.000
Plasma cortisol	1.00	0.30	0.70 (0.62–0.77)	*<0.001*	1.00	0.50	0.50 (0.43–0.57)	*<0.001*
Urine cortisol:creatinine ratio	1.00	1.00	0.00 (−0.08 to 0.08)	1.000	0.89	1.00	−0.11 (−0.17 to −0.04)	*0.003*

Fatigue score (left) and steps per day (right)

Italic values indicate significance of p value (p < 0.05)

*CFS* chronic fatigue syndrome, *CI* confidence interval, *DC* degree centrality

* t test

## Discussion

The most important findings of this study are (a) That there are different interrelations between hormones of the HPA axis, the SAM system, and the thyroid system in CFS patients and healthy controls; and (b) That there is an association between hormone control characteristics and important clinical variables in the CFS group. Thus, based upon a complex statistical approach, the present study provide further evidence that altered neuroendocrine control mechanisms might be at the core of CFS pathophysiology.

A central characteristic of the previous documented attenuation of the HPA axis in CFS is reduced *responsivity*, resulting in weakened cortisol response to common daily stressors (such as awakening), and a flattened cortisol diurnal curve [7]. On the other hand, increased responsivity is a hallmark of the sympathetic cardiovascular control alterations in CFS, causing for instance an exaggerated heart rate and peripheral resistance response during orthostatic challenge [12, 14]. The present results corroborate these previous observations. The plasma hormone concentrations are “snap-shots” from the different endocrine systems, whereas the urine hormone:creatinine ratios, analyzed in morning spot samples, might be seen as an integral of the endocrine activity during the preceding night. A linear association between plasma and urine level of the same hormone directly suggest low plasma variations, possibly explaining the significant relationship among plasma cortisol and urine cortisol:creatinine in the CFS patients, as well as the significant relationship among plasma norepinephrine and urine norepinephrine:creatinine in the healthy controls.

Normally, plasma cortisol is controlled by plasma ACTH [46], explaining the linear relationship between these two hormones in CFS patients as well as controls. However, the regression coefficient is higher in CFS patients, and the interaction with thyroid hormones is strikingly different from the healthy control group, suggesting an alteration in control mechanisms. The network analyses might be interpreted in the same way: The single hormone degree centrality among CFS patients suggests less variability and “tighter” control of the HPA axis, and more variability and “looser” control of the sympathetic nervous system, as compared to healthy controls.

On a general level, the findings in this study complies with the findings of Fuite and co-workers, who reported that adult CFS patients and healthy controls displayed quite similar centralized network parameters, but that there were significant differences in single node centrality indices [19]. However, Fuite and co-wokers found a decrease in plasma cortisol and an increase in plasma norepinephrine degree centrality, as opposed to the present results. The reasons for these discrepancies are not clear; however, results are not necessarily comparable across the two studies, as the total number of nodes in the network analyses was largely different.

Within the CFS group, important clinical variables are associated with network parameters but not with single hormone levels. These findings seem to suggest that the underlying disease mechanisms of CFS are more related to altered neuroendocrine control than to altered hormone levels *per se*. Plasma cortisol degree centrality is of particular interest: Here, the least disabled group of patients are more similar to healthy controls than the most disabled group. This observation is in line with other reports linking altered HPA axis physiology to symptoms and function [9, 11]. Further research should aim at uncover the underlying mechanisms; one promising field of study might be epigenetic alterations of the glucocorticoid receptor gene [47].

Taken together, the findings of this study comply with the “sustained arousal” model of CFS [17]. In this model, a maladaptive stress response is considered a central pathophysiological element, eliciting autonomic and neuroendocrine alterations that parallel the pathophysiology of chronic post-traumatic stress disorder (PTSD). Interestingly, the combination of HPA attenuation and SAM enhancement seem to be a central characteristic of PTSD [48].

### Study strengths and limitations

This study is based upon a large and well-characterized cohort of adolescent CFS, and applies state-of-the-art statistical methods to explore complex interactions among several variables. We did not apply dynamic testing, such as CRH stimulation test or dexamethasone suppression test. Such testing might have yielded increased insight into neuroendocrine control mechanisms, and might also have provided validation of the network analyses. Our wide inclusion criteria might possibly obscure important differences across subgroups; however, subgrouping according to stricter diagnostic definitions did not reveal differential effects. Although the different methods used in this study in general have well-established reliability and validity, these properties have hardly been specifically explored in adolescent CFS patient, and the design did not allow us to do so in the present study either.

## Conclusion

This study reveals different interrelation between hormones of the HPA axis, the SAM system, and the thyroid system in CFS patients and healthy controls, and an association between hormone control characteristics and important clinical variables in the CFS group. These results add to the growing insight of CFS disease mechanisms.

## Abbreviations

CFS: chronic fatigue syndrome
ACTH: adrenocorticotrophic hormone
TSH: thyroid stimulating hormone
FT4: free thyroxine
HPA axis: hypothalamus–pituitary–adrenal axis
SAM system: sympathetic/adrenal medulla system
