# Erratum to: Altered neuroendocrine control and association to clinical symptoms in adolescent chronic fatigue syndrome: a cross-sectional study

**DOI:** 10.1186/s12967-017-1261-1

**Published:** 2017-07-18

**Authors:** Vegard Bruun Wyller, Valeria Vitelli, Dag Sulheim, Even Fagermoen, Anette Winger, Kristin Godang, Jens Bollerslev

**Affiliations:** 1 Division of Medicine and Laboratory Sciences, Medical Faculty, University of Oslo, Oslo, Norway; 2 Department of Paediatrics, Akershus University Hospital, Nordbyhagen, 1478 Lørenskog, Norway; 3 Department of Biostatistics, Institute of Basic Medical Sciences, Oslo Centre for Biostatistics and Epidemiology, University of Oslo, Oslo, Norway; 4 Department of Paediatrics, Oslo University Hospital, Oslo, Norway; 5 Department of Paediatrics, Lillehammer County Hospital, Lillehammer, Norway; 6 Institute of Clinical Medicine, Medical Faculty, University of Oslo, Oslo, Norway; 7 Department of Anesthesiology and Critical Care, Oslo University Hospital, Oslo, Norway; 8 Institute of Nursing Sciences, Oslo and Akershus University College of Applied Sciences, Oslo, Norway; 9 Section of Specialized Endocrinology, Department of Endocrinology, Oslo University Hospital Rikshospitalet, Oslo, Norway

## Erratum to: J Transl Med (2016) 14:121 DOI: 10.1186/s12967-016-0873-1

In the original version of this article [1], published on 5 May 2016, the name of author ‘Valeria Vitelli’ was wrongly displayed.

Originally the author name has been published as:

- Valieria Vitelli

The correct author name is:

- Valeria Vitelli

## References

[1] Wyller VB, Vitelli V, Sulheim D, Fagermoen E, Winger A, Godang K, Bollerslev J (2016). Altered neuroendocrine control and association to clinical symptoms in adolescent chronic fatigue syndrome: a cross-sectional study. J Transl Med.

